# Association of serum pepsinogens and gastrin-17 with *Helicobacter pylori* infection assessed by urea breath test

**DOI:** 10.3389/fcimb.2022.980399

**Published:** 2022-08-16

**Authors:** Jun-peng Zhou, Chang-hai Liu, Bo-wen Liu, You-juan Wang, Mohammed Benghezal, Barry James Marshall, Hong Tang, Hong Li

**Affiliations:** ^1^West China Marshall Research Center for Infectious Diseases, Center of Infectious Diseases, West China Hospital, Sichuan University, Chengdu, China; ^2^Division of Infectious Diseases, State Key Laboratory of Biotherapy and Center of Infectious Diseases, West China Hospital, Sichuan University, Chengdu, China; ^3^Dental Department, 363 Hospital, Chengdu, China; ^4^Health Management Center, West China Hospital of Sichuan University, Chengdu, China; ^5^Helicobacter pylori Research Laboratory, School of Biomedical Sciences, Marshall Centre for Infectious Disease Research and Training, University of Western Australia, Nedlands, WA, Australia; ^6^School of Biomedical Engineering, Marshall Laboratory of Biomedical Engineering, Shenzhen University Health Science Center, Shenzhen, China

**Keywords:** *Helicobacter pylori*, pepsinogen, gastrin-17, urea breath test, gastric cancer

## Abstract

**Background:**

Association of gastric atrophy or cancer with levels of serum pepsinogens, gastrin-17 and anti-*Helicobacter pylori* IgG antibody have been extensively studied. However, the association of serum pepsinogen and gastrin-17 with *H. pylori* infection has not been studied in a large population.

**Aim:**

To investigate the impact of *H. pylori* infection on serum levels of pepsinogens and gastrin-17.

**Methods:**

A total of 354, 972 subjects who underwent health check-ups were included. Serum levels of pepsinogens and gastrin-17 were measured using the enzyme-linked immunosorbent assay*. H. pylori* infection was detected using ^14^C-urea breath test (UBT). Multivariable logistic regression analysis was used to investigate the association of serum pepsinogen and gastrin-17 with *H. pylori* infection.

**Results:**

*H. pylori* prevalence was 33.18% in this study. The mean levels of pepsinogens and gastrin-17 were higher, while the mean pepsinogen-I/II ratio were lower among *H. pylori*-positive than -negative subjects. In *H. pylori*-positive subjects, pepsinogen and gastrin-17 levels correlated positively, whereas the pepsinogen-I/II ratio correlated negatively with UBT values (e.g., the mean serum level of pepsinogen-I in subjects with UBT values in the range of 100-499dpm, 500-1499dpm, and ≥1500dpm was 94.77 ± 38.99, 102.77 ± 43.59, and 111.53 ± 47.47 ng/mL, respectively). Compared with *H. pylori*-negative subjects, the adjusted odds ratio (aOR) of having pepsinogen-I ≤ 70 ng/mL in the three *H. pylori*-positive but with different UBT value groups was 0.31 (*p*<0.001), 0.16 (*p*<0.001), and 0.08 (*p*<0.001), respectively; while the aOR of having G-17>5.70 pmol/L was 4.56 (*p*<0.001), 7.43 (*p*<0.001), and 7.12 (*p*<0.001). This suggested that *H. pylori*-positive subjects with higher UBT values were less likely to have pepsinogen-I ≤70 ng/mL (a serum marker for gastric atrophy), but more likely to have gastrin-17 >5.70 pmol/L (a marker for peptic ulcer).

**Conclusions:**

*H. pylori*-positive subjects with higher UBT values are unlikely to have gastric atrophy, but may have greater risk of severe gastritis or peptic ulcers. Our study suggests that *H. pylori*-positive patients with high UBT values may benefit the most from *H. pylori* eradication.

## Introduction

*Helicobacter pylori* is one of the most common human bacterial pathogens, infecting approximately half of the world’s population (Hooi et al., 2017). Chronic *H. pylori* infection, usually acquired in childhood, causes active gastritis in all infected subjects (Chey et al., 2017; Malfertheiner et al., 2017). Without antibiotic treatment, *H. pylori* gastritis can progress to gastric atrophy, intestinal metaplasia, dysplasia, and eventually gastric cancer (Sugano et al., 2015; Malfertheiner et al., 2017; Liu et al., 2018). With more than 1 million new cases diagnosed in 2020 and approximately 0.77 million deaths, gastric cancer is the fifth most frequent cancer and the fourth leading cause of cancer death worldwide (Sung et al., 2021).

The non-invasive serological biopsy including 5 stomach-specific circulating biomarkers (serum pepsinogen I, PG-I; PG-II; the PG-I/PG-II ratio; gastrin-17, G-17; and anti-*H. pylori* IgG antibody) have been widely used in clinical practice for the assessment of gastric atrophy and gastric cancer risk (Bodger et al., 2001; Miki and Urita, 2007; Miki, 2011; Tu et al., 2017; Cai et al., 2019). PG-I is secreted mainly by chief cells and neck mucous cells in the gastric corpus and fundus glands, while PG-II is secreted not only by the fundus glands but also by the pyloric glands of the gastric antrum and the Brunner’s glands of the proximal duodenum. The serum PG-I decreases with the atrophy of the gastric fundus, while the PG-II level remains relatively constant. Thus, low serum PG-I level (≤70 ng/ml) and/or low PG-I/PG-II ratio (≤3.0) are usually used to indicate the chronic atrophic gastritis in the fundus, a high risk factor for the development of gastric cancer (Miki et al., 1993; Kitahara et al., 1999; Tu et al., 2017). Of note, the PGs have been used in clinical practice as biomarkers for chronic atrophic gastritis even before the discovery of *H. pylori* (Miki and Urita, 2007; Miki, 2011).

Serum G-17 is the amidated and biologically active gastrin secreted by the G cells in the gastric antrum. It plays an important role in gastric physiology by stimulating acid secretion (mainly in response to low acidity in the stomach) and maintaining gastric mucosal homeostasis (Watson et al., 2006). It has been suggested that an elevated intragastric pH induced by chronic atrophic gastritis in the corpus may cause hypersecretion of G-17 from the antrum (Fourmy et al., 2011). G-17 is also a pro-proliferative and anti-apoptotic hormone which has been proposed to play an important role in gastric carcinogenesis (Watson et al., 2006; Takaishi et al., 2009). It has been reported that the serum G-17 level in patients with gastric cancer is higher than those without (Sun et al., 2014; Cai et al., 2019; Shen et al., 2021). Thus, elevated serum G-17 level is commonly used to identify individuals at high risk of developing gastric cancer (Zagari et al., 2017; Cai et al., 2019).

There are numerous studies that have assessed the different combination of PG-I, PG-II, PG-I/PG-II ratio, G-17 and anti-*H. pylori* IgG antibody as independent risk factors in screening patients with high risk of gastric cancer (Miki et al., 1993; Miki and Urita, 2007; Miki, 2011; Tu et al., 2017; Zagari et al., 2017; Cai et al., 2019; Shen et al., 2021). *H. pylori* infection is the most important risk factor for gastric cancer, as nearly 90% of gastric cancer cases can be ascribed to this gastric bacterium (Dang and Graham, 2017; Malfertheiner et al., 2017). However, studies specifically investigating the association of serum PGs and G-17 with *H. pylori* infection are scarce. Here, with the inclusion of 354, 972 subjects who underwent a health checkup in West China Hospital, we investigated the effects of *H. pylori* infection (assessed by ^14^C urea breath test, UBT) on serum PGs and G-17.

## Materials and methods

### Subjects

A total of 455,733 subjects who underwent medical examination at the Physical Examination Centre of West China Hospital, Sichuan University between January 2014 and December 2017 were investigated. Subjects who underwent UBT and aged 18 years or older were included. Subjects with incomplete data and aged less than 18 years were excluded. If the included participants had more than one time of health check-up records, only the first-time medical record was used for this cross-sectional study. A total of 354, 972 subjects were included in the final analysis. The study was performed in accordance with the ethical guidelines in the Declaration of Helsinki and the International Conference on Harmonization Guidelines for Good Clinical Practice. The study was approved by the Ethics Committee of West China Hospital of Sichuan University. All participants provided informed consent.

### Data collection

The data was automatically extracted using a Hospital Information System (HIS). Using calibrated instruments with standard protocols, a range of physical measurements including height, weight, hip and waist circumferences and blood pressure etc. were recorded by trained technicians. The body mass index (BMI) of the subjects was calculated by dividing the subject’s weight (kg) by the square of their height (m^2^). A BMI <24 kg/m^2^ was defined as normal weight, whereas a BMI in the range of 24-27.9 kg/m^2^ was considered as overweight, and a BMI ≥28 kg/m^2^ was defined as obese (Zhou, 2002). For each subject, overnight (12 h) fasting blood samples were taken for routine biochemical analyses including complete blood count, serum levels of alanine aminotransferase (ALT), aspartate aminotransferase (AST), γ-glutamyl transpeptidase (GGT), high-density lipoprotein cholesterol (HDL-C), low-density lipoprotein cholesterol (LDL-C), fasting plasma glucose (FPG), triglycerides (TG), total cholesterol, creatinine, bilirubin, and platelets.

Metabolic syndrome was defined as having central obesity (waist circumference ≥ 90 cm in men or ≥80 cm in women) plus any two of the following four factors: 1) raised triglyceride, defined by triglyceride levels ≥1.7 mmol/L (150 mg/dL) or use of lipid-lowering agents; 2) reduced HDL-C, defined by HDL-C < 1.03 mmol/L (40 mg/dL) in men or <1.29 mmol/L (50 mg/dL) in women or use of lipid-lowering agents; 3) raised blood pressure, determined by blood pressure ≥130/85 mm Hg or use of blood pressure-lowering agents; 4) raised FPG, defined by FPG level ≥ 5.6 mmol/L (100 mg/dL) or previously diagnosed type 2 diabetes or use of blood glucose-lowering agents (Alberti et al., 2006; Alberti et al., 2009).

The presence of *H. pylori* infection was detected using the ^14^C UBT (Shenzhen Zhonghe Headway Bio-Sci & Tech Co. Ltd., Shenzhen, Guangdong, China). Subjects with the measured disintegrations per minute (dpm) value ≥ 100 were diagnosed as having *H. pylori* infection (Bielański et al., 1996). Positive *H. pylori* infected subjects were further divided into three groups based on UBT values in the range of 100-499 dpm, 500-1499 dpm, and ≥1500 dpm, respectively.

Serum G-17, PGI, and PGII were measured using the enzyme-linked immunosorbent assay according to the manufacturer’s instructions. Abnormal serum PGs and G-17 were defined as PGI ≤ 70 ng/mL, PGII>37.23 ng/mL, PGI/PGII ≤ 3 and G-17>5.70 pmol/L (Miki et al., 1993; Miki and Urita, 2007; Miki, 2011; Bang et al., 2019; Cai et al., 2019).

### Statistical analysis

Data were reported as the mean ± standard deviation for normal and median (interquartile range) for non-normal continuous variables, while frequency was used for discrete variables. In the univariate comparisons, we used the Student’s t-test and ANOVA with Bonferroni adjustments for continuous samples and chi-square test or Fisher’s exact test for the qualitative ones. Non-parametric alternatives (Mann–Whitney U and Kruskal-Wallis tests) were used for non-normal distributions.

Pearson’s correlation test was performed to determinate the correlation of serum levels of PGs, PGI/PGII ratio, G-17 with UBT values, age, sex, and BMI. Logistic regression models were used to estimate adjusted odds ratios (aORs) of specific disease prevalence associated with outcome. Covariates were selected for analysis according to their biologically plausible potential to act as confounders or predictors for each outcome. The potential predictors were as follows: age, gender, location (rural or urban), year of health check-up year, BMI, *H. pylori* infection. The collinearity between factors included in the multivariable analyses was checked by using variance inflation factor (VIF) and tolerance (1/VIF) values. Variables with very high VIF values indicating possible redundancy entered into different multivariable models.

The method used for missing data was complete-case analysis since statistical packages excluded individuals with any missing value. All CIs, significance tests, and resulting *p* values were 2-sided, values were supposed to be statistically significant when *p <*0.05. Statistical analysis was performed using STATA (version 14) and PRISM (version 8).

## Results

### The prevalence of *H. pylori* infection was different among different age groups

**Figure 1:**
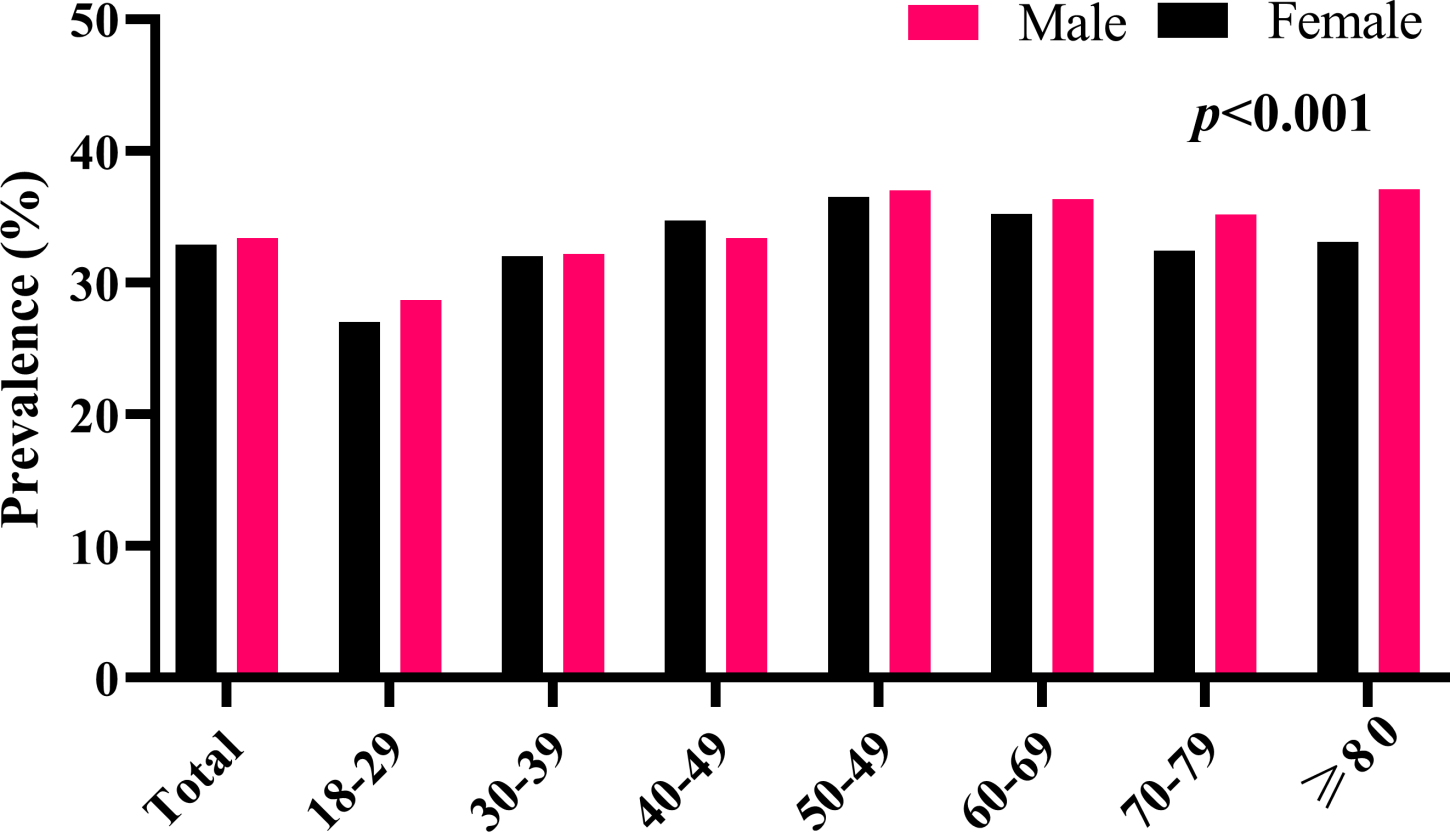
The prevalence of *H. pylori* infection among different age groups.

A total of 354, 972 subjects (203,384 males, and 151,588 females) with a mean age of 43.80 ± 12.44 years were included in the final analysis. The overall prevalence of *H. pylori* infection was 33.18% (117,805/354,972) (**Table 1**). The prevalence of *H. pylori* infection in males and females was 33.42% (67,964/203,384) and 32.88% (49,841/151,588), respectively. Interestingly, among the 8 different age groups (18-29, 30-39, 40-49, 50-59, 60-69, 70-79, and ≥80 years), the prevalence of H. pylori infection in subjects aged between 18-29 years was the lowest in both males and females (27.86% and 28.69%), which gradually increased to 36.79% and 36.97% in subjects aged between 50-59 years (**Figure 1**).

**Table 1:** Characteristics of subjects in *H. pylori* positive and negative groups.

	*H. pylori* positive	*H. pylori* negative	*p* value
n (%)	117805 (33.18%)	237167 (66.81%)	
Age (years)	44.76 ± 12.28	43.33 ± 12.50	<0.001
Male (%)	57.69%	57.10%	0.001
Urban resident (%)	49.30%	51.80%	<0.001
BMI (kg/m^2^)	23.69 ± 3.28	23.46 ± 3.26	<0.001
Normal (<24) (%)	64.25%	62.70%	<0.001
Overweight (24-27.9) (%)	27.74%	29.59%	<0.001
Obese (≥28) (%)	2.95%	3.46%	<0.001
Waist circumference (♂) (cm)	84.81 ± 8.54	85.25 ± 8.50	<0.001
Waist circumference (♀) (cm)	73.15 ± 8.11	73.81 ± 8.18	<0.001
Hip circumference (♂) (cm)	96.31 ± 5.52	96.35 ± 5.59	<0.001
Hip circumference (♀) (cm)	91.88 ± 5.27	92.11 ± 5.35	0.088
Waist-to-hip ratio	0.84 ± 0.05	0.85 ± 0.05	<0.001
Biochemistry			
Triglycerides (mg/dL)	1.57 ± 0.36	1.58 ± 0.36	<0.001
Total cholesterol (mg/dL)	4.85 ± 0.22	4.87 ± 0.22	<0.001
Creatinine (mg/dL)	70.61 ± 10.90	70.90 ± 10.86	<0.001
Bilirubin (mmol/L)	14.53 ± 1.20	14.54 ± 1.20	<0.001
AST(U/L)	25.31 ± 2.65	25.42 ± 2.64	<0.001
ALT (U/L)	27.61 ± 7.14	27.54 ± 7.14	0.007
GGT (U/L)	32.82 ± 11.69	33.11 ± 11.66	<0.001
HDL-C (mg/dL)	1.45 ± 0.18	1.45 ± 0.18	0.352
LDL-C (mg/dL)	2.76 ± 0.19	2.78 ± 0.18	<0.001
Platelets (*10^9^/L)	194.61 ± 14.01	193.25 ± 13.80	<0.001
Metabolic syndrome (yes %)	12.55%	14.33%	<0.001
WC >90 cm (♂) or >80 cm (♀) (%)	24.97%	26.88%	<0.001
Triglycerides >1.7mmol/L (%)	28.75%	29.89%	<0.001
HDL-C <40 mg/dL (♂) or <50 mg/dL (♀) (%)	17.46%	17.78%	<0.001
Blood pressure ≥130/85 mmHg (%)	12.25%	14.20%	<0.001
FPG≥100 mg/dL (%)	16.53%	19.03%	<0.001
Diabetes mellitus (%)	3.95%	4.93%	<0.001
PG-I	97.08 ± 40.53	75.81 ± 33.30	<0.001
PG-II	14.84 ± 8.94	8.11 ± 5.93	<0.001
PG-I ≤ 70 (%)	24.20%	51.60%	<0.001
PG-II>37.23 (%)	3.10%	0.70%	<0.001
PG-I/PG-II	7.65 ± 5.03	10.84 ± 4.56	<0.001
PG-I/PG-II ≤ 3 (%)	2.17%	1.43%	<0.001
G-17 (pmol/L)	8.11 ± 9.23	4.12 ± 7.72	<0.001
G-17 >5.7 (%)	48.40%	15.20%	<0.001

BMI, body mass index; AST, aspartate aminotransferase; ALT, alanine aminotransferase; GGT, γ-glutamyl transpeptidase; HDL-C, high-density lipoprotein cholesterol; LDL-C, low-density lipoprotein cholesterol; WC, Waist circumference; FPG, fasting plasma glucose; PGI, Pepsinogen I; PGII, Pepsinogen II; G-17, gastrin-17.

**Figure 2:**
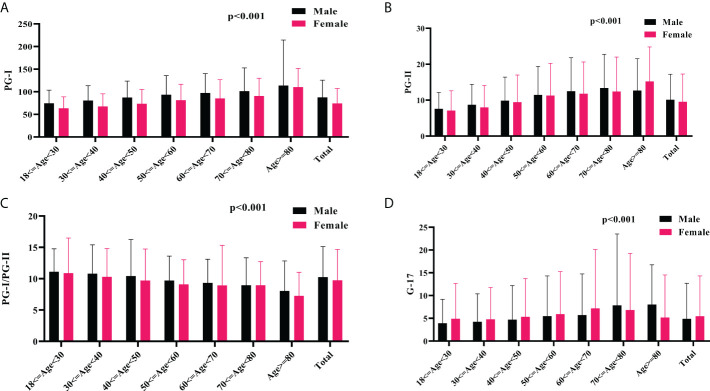
The association of serum PGs and G-17 with age and gender. The mean serum levels of PGI **(A)**, PGII **(B)**, PGI/PGII ratio **(C)**, and G-17 **(D)** among different gender and age groups. PG, pepsinogen; G-17, gastrin-17.

Of note, the mean serum levels of PGs, and G-17 increased, whereas the PGI/PGII ratio decreased gradually with age. Overall, the male subjects had higher serum levels of PGs and PGI/PGII ratio, whereas lower serum level of G-17 than that of their female counterparts (**Figure 2**). Based on Pearson’s correlation test, there was a positive correlation between age and PGI (*r*=0.196, *p*<0.001), PGII (*r*= 0.204, *p*<0.001), and G-17 (*r*=0.078, *p*<0.001); whereas a negative correlation between age and PGI/PGII ratio (*r*=-0.114, *p*<0.001). There was a positive correlation between male gender and PGI (*r*=0.183, *p*<0.001), PGII (*r*= 0.038, *p*<0.001), and PGI/PGII ratio (*r*=0.053, *p*<0.001), whereas a negative correlation between male gender and G-17 (*r*=-0.035, *p*<0.001). Interestingly, the Pearson’s correlation test revealed a positive correlation between BMI and serum levels of PGI (*r*=0.016, *p*=0.015), and PGII (*r*=0.018, *p*=0.005).

### Subjects with *H. pylori* infection have higher serum levels of PGs and G-17, but lower PGI/PGII ratio than those without

The mean PGI and PGII levels in *H. pylori*-positive subjects were 97.08 ± 40.53 and 14.84 ± 8.94 ng/mL, which were much higher than the 75.81 ± 33.30 and 8.11 ± 5.93 ng/mL in *H. pylori*-negative subjects (P<0.001) (**Table 1**). The percentage of individuals having PGI ≤ 70 ng/mL was much lower in *H. pylori*-positive subjects than that of the *H. pylori*-negative subjects (24.20% vs 51.60%, *p*<0.001), whereas the percentage of individuals having PGII≤37.23 ng/mL among *H. pylori*-positive subjects was higher than that of the *H. pylori*-negative subjects (3.10% vs 0.70%, *p*<0.001). The mean PGI/PGII ratio in the *H. pylori*-positive subjects was 7.65 ± 5.03, which was much lower than the mean ratio of 10.84 ± 4.56 in *H. pylori*-negative subjects (*p*<0.001). The percentage of subjects with PGI/PGII ≤ 3 in *H. pylori*-positive and *H. pylori*-negative subjects was 2.17% and 1.43%, respectively (**Table 1**).

The mean serum G-17 level in the *H. pylori*-positive subjects was 8.11 ± 9.23 pmol/L, which was much higher than the mean value of 4.12 ± 7.72 pmol/L in the *H. pylori*-negative subjects (*p*<0.001) (**Table 1**). The percentage of individuals having G-17 >5.7 among *H. pylori*-positive subjects was higher than that of the *H. pylori*-negative subjects (48.40% vs 15.20%, *p*<0.001).

### *H. pylori*-positive subjects with higher UBT values have higher serum levels of PGs but lower PGI/II ratio

**Figure 3:**
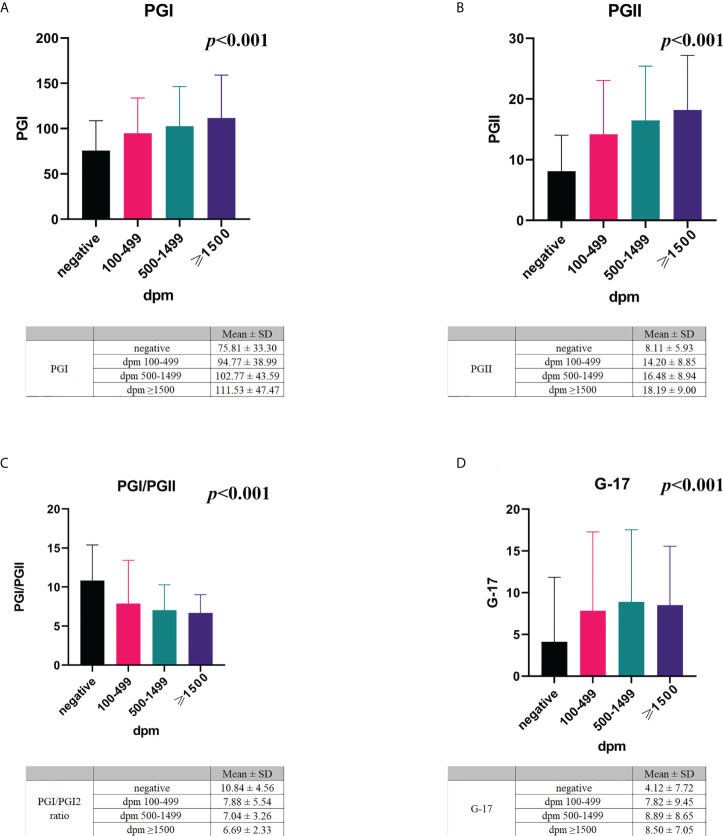
The association of serum PGs and G-17 with *H*. *pylori* infection. The mean serum levels of PGI **(A)**, PGII **(B)**, PGI/PGII ratio **(C)**, and G-17 **(D)** in *H*. *pylori*-negative and -positive subjects with different UBT values. PG, pepsinogen; G-17, gastrin-17; dpm, disintegrations per minute.

Among the *H. pylori*-positive subjects with different UBT values, we analysed whether the serum levels of PGs and G-17, and the PGI/II ratio were different. We demonstrated that the mean PGI levels in *H. pylori*-positive subjects with UBT values in the range of 100-499 dpm, 500-1499 dpm, and ≥1500 dpm were 94.77 ± 38.99, 102.77 ± 43.59, and 111.53 ± 47.47 ng/mL, respectively (**Figure 3A**). The mean PGII level among the three different UBT value groups was 14.20 ± 8.85, 16.48 ± 8.94, and 18.19 ± 9.00 ng/mL, respectively (**Figure 3B**). The PGI/PGII ratio in the three different UBT value groups was 7.88 ± 5.54, 7.04 ± 3.26, and 6.69 ± 2.33, respectively (**Figure 3C**). The serum G-17 levels in the three groups were 7.82 ± 9.45, 8.89 ± 8.65, and 8.50 ± 7.05 pmol/L, respectively (**Figure 3D**).

The Pearson’s correlation test revealed a positive correlation between UBT values and serum levels of PGI (*r*=0.232, *p*<0.001), PGII (*r*=0.352, *p*<0.001), and G-17 (*r*=0.178, *p*<0.001), whereas a negative correlation between PGI/PGII ratio and the UBT values (*r*=-0.241, *p*<0.001).

### *H. pylori*-positive subjects with higher UBT values are less likely to have PGI ≤ 70 ng/mL, but more likely to have G-17>5.70 pmol/L

Multivariable analysis was carried out to investigate whether *H. pylori*-positive subjects with different UBT values had different effects on the abnormalities of serum PGs and G-17. After adjustment for sociodemographic characteristics including age, sex, region (urban or rural), year of health check-up, and BMI, we demonstrated that compared with *H. pylori*-negative subjects, the aOR of having PGI ≤ 70 ng/mL in *H. pylori*-positive subjects with UBT values in the range of 100-499 dpm, 500-1499 dpm, and ≥1500 dpm was 0.31 (95% CI 0.29-0.33, *p*<0.001), 0.16 (95% CI 0.14-0.18, *p*<0.001), and 0.08 (95% CI 0.04-0.15, *p*<0.001), respectively; and the aOR of having G-17>5.70 pmol/L was 4.56 (95% CI 4.24-4.91, *p*<0.001), 7.43 (95% CI 6.67-8.28, *p*<0.001), and 7.12 (95% CI 4.66-10.88, *p*<0.001) (**Table 2**). This suggested that *H. pylori*-positive subjects with higher UBT values were less likely to have PGI ≤ 70 ng/mL, but more likely to have G-17>5.70 pmol/L.

**Table 2:** Adjusted ORs for abnormalities of serum PGs and G-17 in *H. pylori*-positive subjects with different UBT values.

UBT values	*H. pylori* negative	*H. pylori* positive							
		PGI ≤ 70		PGII>37.23		PGI/PGII ≤ 3		G-17>5.70	
		aOR (95% CI)	*p* value	aOR (95% CI)	*p* value	aOR (95% CI)	*p* value	aOR (95% CI)	*p* value
Dpm≥100	Reference	0.26 (0.24-0.28)	< 0.001	4.59 (3.65-5.78)	< 0.001	1.53 (1.24-1.88)	< 0.001	5.20 (4.87-5.55)	<0.001
-Dpm (100-499)	Reference	0.31 (0.29-0.33)	<0.001	4.57 (3.57-5.86)	<0.001	1.24 (0.93-1.66)	0.143	4.56 (4.24-4.91)	<0.001
-Dpm (500-1499)	Reference	0.16 (0.14-0.18)	<0.001	4.69 (3.38-6.52)	<0.001	0.64 (0.36-1.12)	0.120	7.43 (6.67-8.28)	<0.001
-Dpm (≥1500)	Reference	0.08 (0.04-0.15)	<0.001	4.20 (1.29-13.64)	0.017	1.56 (0.37-6.46)	0.543	7.12 (4.66-10.88)	<0.001

Logistic regressions were stratified by sex, region, year of health check-up, and adjusted for age and BMI.

PG, pepsinogen; G-17, gastrin-17; aOR, adjusted odds ratio.

### The abnormalities of serum PGs and G-17 are associated with age and gender

As age and gender are associated with risk of many cancers, multivariable logistic regression analysis was performed to investigate whether different age and gender had different effects on the abnormalities of serum PGs and G-17. We demonstrated that *H. pylori*-positive subjects aged less than 40 years had a lower chance to have PGI ≤ 70 ng/mL than those *H. pylori*-positive subjects aged 40 years and older (aOR 0.19 vs aOR 0.29, *p*<0.001), but a higher chance to have G-17>5.70 pmol/L (aOR 6.27 vs aOR 4.87, *p*<0.001). Similarly, our results demonstrated that female *H. pylori*-positive subjects were less likely to have PGI ≤ 70 ng/mL than their male counterparts (aOR 0.18 vs aOR 0.38, *p*<0.001), but more likely to have G-17>5.70 pmol/L (aOR 5.90 vs aOR 4.62, *p*<0.001) (**Table 3**).

**Table 3:** Subgroup analysis of adjusted ORs for abnormalities of serum PGs and G-17 in *H. pylori*-positive subjects.

	PGI ≤ 70		PGII>37.23		PGI/PGII ≤ 3		G-17>5.70	
Subgroup	aOR (95% CI)	*p* value	aOR (95% CI)	*p* value	aOR (95% CI)	*p* value	aOR (95% CI)	*p* value
**Age**								
Age<40	0.19 (0.22-0.17)	<0.001	2.54 (1.33-4.87)	0.005	1.99 (1.11-3.59)	0.211	6.27 (5.54-7.10)	<0.001
Age>=40	0.29 (0.27-0.32)	<0.001	4.69 (3.68-5.98)	<0.001	1.49 (1.19-1.87)	<0.001	4.87 (4.49-5.24)	<0.001
**Gender**								
Female	0.18 (0.16-0.20)	<0.001	4.35 (3.18-5.95)	<0.001	1.44 (1.09-1.90)	0.009	5.90 (5.37-6.48)	<0.001
Male	0.38 (0.34-0.41)	<0.001	4.65 (3.37-6.41)	<0.001	1.72 (1.24-2.38)	0.001	4.62 (4.22-5.06)	<0.001
**Location**								
Urban	0.27 (0.25-0.29)	<0.001	4.66 (3.52-6.19)	<0.001	1.67 (1.28-2.18)	<0.001	5.13 (4.72-5.57)	<0.001
Rural	0.24 (0.21-0.26)	<0.001	4.20 (2.90-6.09)	<0.001	1.34 (0.94-1.91)	0.111	5.35 (4.81-5.95)	<0.001

Logistic regression was stratified by sex, region, year of health check, and adjusted for age and BMI.

PG, pepsinogen; G-17, gastrin-17; aOR, adjusted odds ratio.

## Discussion

This study included a large health checkup population (354, 972 subjects) to analyze the impact of *H. pylori* infection on serum PGI and PGII levels, PGI/PGII ratio, and G-17 levels. In *H. pylori* positive subjects, the serum PGI, PGII, and G-17 levels were much higher, while the PGI/PGII ratio was much lower than that of the *H. pylori*-negative subjects. We further demonstrated a positive correlation between the levels of PGI and PGII and the UBT values, while a negative correlation between the PGI/PGII ratio and the UBT values. Multivariable analysis demonstrated that *H. pylori*-positive subjects with higher UBT values were more likely to have G-17>5.70 pmol/L, but less likely to have PGI ≤ 70 ng/mL. Compared with older and male *H. pylori*-positive subjects, younger and female *H. pylori*-positive subjects are less likely to have PGI ≤ 70 ng/mL.

In the present study, we demonstrated that the prevalence of *H. pylori* infection was 33.18% in this study, which was much lower than the previously reported *H. pylori* prevalence rate of approximately 50% in China (Hooi et al., 2017; Wang et al., 2019; Xiong et al., 2020). The relatively low prevalence of *H. pylori* infection in this study and also in another recent study (27.5%) (Shu et al., 2019) might be partly explained by the fact that the subjects were recruited in the capital city of the province where the economic and educational levels are higher than the general population (Nagy et al., 2016; Leja et al., 2019). It has been reported that *H. pylori* infection prevalence in urban populations was much lower the rural populations, as the improved living standards associated with urbanization play an important role in reducing the horizontal transmission of *H. pylori* (Nagy et al., 2016). It is expected that the prevalence of *H. pylori* infection will continue to decrease with sustainable urbanization in China.

*H. pylori* infection is considered to be the most important etiological factor for gastric cancer (Malfertheiner et al., 2017; El-Serag et al., 2018; Liu et al., 2018). It is now well-accepted that *H. pylori* infection is the crucial and initiating factor in the Correa Cascade model of gastric cancer: *H. pylori* infection→acute gastritis→chronic gastritis→gastric atrophy→intestinal metaplasia→dysplasia→gastric cancer (Malfertheiner et al., 2017). Previous studies have shown significant associations between *H. pylori* infection and elevated levels of PGI, PGII, and G-17, and reduced PG-I/PG-II ratio in *H. pylori*-related non-atrophic chronic gastritis (Asaka et al., 1992; Bodger et al., 2001; Ohkusa et al., 2004; Miki and Urita, 2007; Miki, 2011; Gong et al., 2014). It has been reported that *H. pylori*-mediated gastrin induction is type IV secretion system (T4SS)-dependent (Rieder et al., 2005; Gunawardhana et al., 2017). *H. pylori* infection induces the expression of heparin-binding EGF-like growth factor (HB-EGF) *via* T4SS. Subsequently, the increased HB-EGF binds to the EGF receptor in G cells to sequentially activate C-Raf, Mek-1, and Erk2 in the mitogen-activated protein kinase (MAPK) pathway, resulting in an increased gastrin expression (Gunawardhana et al., 2017). *H. pylori* lipopolysaccharide (LPS) has been reported to be responsible for stimulating pepsinogen secretion in a dose-dependent manner (Young et al., 1992; Young et al., 2002). Both the composition and structure of the LPS molecule play a modulatory role in pepsinogen release. LPS from other gastrointestinal bacterial species including *Helicobacter nemestrinae* and *Campylobacter jejuni* have no effect on pepsinogen secretion (Young et al., 2002).

In this study, we demonstrated a progressive increase in serum PG levels with increasing UBT values among this health check-up population, which is consistent with a previous study (Huang et al., 2016). It has been reported that the UBT values correlate positively with the intragastric *H. pylori* bacterial load (Perri et al., 1998; Zagari et al., 2005). Thus, it can be inferred that the higher the serum PG levels, the denser of *H. pylori* infection in the stomach. As the *H. pylori*-associated gastritis progresses, the gastric mucosa may become inhospitable for the colonization of *H. pylori*, leading to a reduced UBT value or even a negative *H. pylori* serology test (Ohata et al., 2004; Tu et al., 2017). The serum PG I level decreases with the reduced *H. pylori* bacterial load and the loss of fundic gland mucosa (Bodger et al., 2001; Ohata et al., 2004; Miki and Urita, 2007; Tu et al., 2017). It has been reported that in *H. pylori*-infected patients, serum PG-I level positively correlates with the severity of antral inflammation, while a stepwise decrease of serum PG-I level was observed with progression from an antral predominant gastritis, through pangastritis, to a corpus predominant gastritis (Bodger et al., 2001). The lowest serum PG-I level and the PG-I/PG-II ratio were observed in patients with severe corpus atrophy and gastric cancer (Bodger et al., 2001). Hence, the combination of UBT and PG-I might be used in clinical practice to predict the functional and histological status of the gastric mucosa. *H. pylori*-infected patients having high levels of serum PG-I and UBT value may have antral predominant gastritis and high risk of peptic ulcers but low risk of gastric atrophy and cancer. In contrast, *H. pylori*-infected subjects having decreased levels of serum PG-I and UBT values (or even negative UBT) are very likely to have high risk of gastric atrophy and cancer.

Older age and male sex are always associated with a higher risk of gastric cancer (Rawla and Barsouk, 2019; Etemadi, et. al., 2020; Machlowska et al., 2020; Lou et al., 2020; Sung et al., 2021; Wong et al., 2021). The incidence rate of gastric cancer increases progressively with age, especially after 40 years old. In a recently developed gastric cancer risk prediction rule consisting of seven variables (age, sex, PG I/II ratio, G-17, anti-*H. pylori* infection IgG status, pickled food, and fried food), points assigned to age 60-69, and older than 69 were 6 and 9, respectively, which were much higher than the points assigned to other variables (Cai et al., 2019). For males, the age-standardized incidence rate of gastric cancer is approximately twice the rate for females (Rawla and Barsouk, 2019; Etemadi, et. al., 2020; Machlowska et al., 2020; Sung et al., 2021). The lower gastric cancer rate in women might be partly explained by the protective effect of estrogen (Chandanos and Lagergren, 2008; Rawla and Barsouk, 2019). Taking the age and gender into account, it would be expected that the effect of *H. pylori* infection on subjects with different age and gender is different. Indeed, in the present study, we demonstrated that compared with older and male *H. pylori*-positive subjects, younger and female *H. pylori*-positive subjects are less likely to have PGI ≤ 70 ng/mL (**Table 3**).

Our study has several limitations. Firstly, the diagnosis of *H. pylori* infection was based on UBT rather than the gold standard, biopsy/culture. However, biopsy/culture requires endoscopy, which is invasive and usually not included in a routine health check-up package. Although UBT is not the gold standard, the advantages of easy to operate and non-invasive procedure, and also the both high sensitivity and specificity (approximately 95%) make it the best and most frequently used test for the diagnosis of *H. pylori* infection (Malfertheiner et al., 2017; El-Serag et al., 2018). Secondly, *H. pylori* IgG assay was not performed to the health check-up population in this study. Due to the retrospective nature of this study, we were unable to conduct the *H. pylori* IgG assay to compare the results of UBT and *H. pylori* IgG assay in analyzing the effect of *H. pylori* infection on serum pepsinogens and G-17. Thirdly, the information of medication usage such as the proton pump inhibitors and antibiotics was not recorded during the health checkup examination, which may impact the diagnosis of *H. pylori* infection by UBT. Fourthly, this study was a single hospital-based cross-sectional study, so the generated findings may not be generalized to the entire Chinese population. Nevertheless, our study included the largest health checkup population so far (354, 972 subjects) to analyze the association between *H. pylori* infection and serum pepsinogen and gastrin levels.

In summary, we demonstrate that *H. pylori* infection is closely associated with elevated levels of PGs and G-17, but with a reduced PG-I/PG-II ratio among the health checkup population. The serum levels of PGs are positively correlated, while the PGI/PGII ratio is negatively correlated with the intragastric *H. pylori* bacterial load as reflected by the UBT values. *H. pylori*-positive subjects with higher UBT values are less likely to have PGI ≤ 70 ng/mL (a serum marker for gastric atrophy), but more likely to have G-17>5.70 pmol/L (stimulating gastric acid secretion and peptic ulcers), suggesting that *H. pylori*-positive patients with high UBT values are unlikely to have gastric atrophy, but may have a greater risk of severe gastritis and peptic ulcers. Thus, *H. pylori*-positive patients with high UBT values, and high levels of serum PGs and G-17 may benefit the most from *H. pylori* eradication.

## Data availability statement

The raw data supporting the conclusions of this article will be made available by the authors, without undue reservation.

## Ethics statement

The studies involving human participants were reviewed and approved by Ethics Committee of West China Hospital of Sichuan University. The patients/participants provided their written informed consent to participate in this study.

## Author contributions

J-pZ, C-hL, B-wL, and HL wrote the manuscript. MB, BJM, Y-jW, and HT edited the manuscript. All authors contributed to the article and approved the submitted version.

## Funding

This study was supported by National Natural Science Foundation of China (82072248) and 1.3.5 Project for Disciplines of Excellence, West China Hospital, Sichuan University (ZY2016201).

## Conflict of interest

The authors declare that the research was conducted in the absence of any commercial or financial relationships that could be construed as a potential conflict of interest.

## Publisher’s note

All claims expressed in this article are solely those of the authors and do not necessarily represent those of their affiliated organizations, or those of the publisher, the editors and the reviewers. Any product that may be evaluated in this article, or claim that may be made by its manufacturer, is not guaranteed or endorsed by the publisher.
